# Social Information Is Integrated into Value and Confidence Judgments According to Its Reliability

**DOI:** 10.1523/JNEUROSCI.3880-16.2017

**Published:** 2017-06-21

**Authors:** Benedetto De Martino, Sebastian Bobadilla-Suarez, Takao Nouguchi, Tali Sharot, Bradley C. Love

**Affiliations:** ^1^Institute of Cognitive Neuroscience, University College London, WC1N 3AZ London, United Kingdom,; ^2^Alan Turing Institute, British Library, London NW1 2DB, United Kingdom, and; ^3^Department of Experimental Psychology, University College London, London WC1H 0AP, United Kingdom

**Keywords:** Bayesian, confidence, integration, social, value, vmPFC

## Abstract

How much we like something, whether it be a bottle of wine or a new film, is affected by the opinions of others. However, the social information that we receive can be contradictory and vary in its reliability. Here, we tested whether the brain incorporates these statistics when judging value and confidence. Participants provided value judgments about consumer goods in the presence of online reviews. We found that participants updated their initial value and confidence judgments in a Bayesian fashion, taking into account both the uncertainty of their initial beliefs and the reliability of the social information. Activity in dorsomedial prefrontal cortex tracked the degree of belief update. Analogous to how lower-level perceptual information is integrated, we found that the human brain integrates social information according to its reliability when judging value and confidence.

**SIGNIFICANCE STATEMENT** The field of perceptual decision making has shown that the sensory system integrates different sources of information according to their respective reliability, as predicted by a Bayesian inference scheme. In this work, we hypothesized that a similar coding scheme is implemented by the human brain to process social signals and guide complex, value-based decisions. We provide experimental evidence that the human prefrontal cortex's activity is consistent with a Bayesian computation that integrates social information that differs in reliability and that this integration affects the neural representation of value and confidence.

## Introduction

We may not like to admit it, but our own opinions are greatly influenced by those of other people. When we book a holiday, buy a new electronic device, or choose a film to watch, we often rely on the opinions of other people expressed in the forms of reviews. Taking other people's judgments into account can be a sensible strategy for a social species. Humans have similar needs and therefore often share preferences with others in their sociodemographic group. The effect of social influence on judgments (i.e., social conformity) has been a topic of intense investigation (Cialdini and Goldstein, 2004) and, in more recent years, the field of cognitive neuroscience has begun to dissect the circuitry underpinning social conformity (Behrens et al., 2009; Berns et al., 2010; Campbell-Meiklejohn et al., 2010; Klucharev et al., 2011; De Martino et al., 2013b; Izuma and Adolphs, 2013). However, the social information that we receive, much like our own beliefs, varies in its reliability or uncertainty. For example, should one purchase headphones on Amazon's website with a 4-star average based on hundreds of reviews or a competing product with a 5-star average based on only a few people's opinions? In such circumstances, people should be sensitive to the opinions of others but also to their prevalence.

The aim of the current study was to investigate whether the human brain integrates social information according to its reliability and how this in turn affects valuation and confidence judgments. More specifically, we evaluated whether people integrate their initial beliefs and those of others in a Bayesian fashion such that the combination is weighted by the uncertainty of each source of information. For example, according to the Bayesian view, in the example above, people should update their beliefs most toward the social consensus when they are initially uncertain about the value of the headphones and there are a large number of Amazon reviewers.

Bayesian inference is a normative framework for how prior beliefs are updated in light of new information (Vilares and Kording, 2011; O'Reilly et al., 2012). One empirical signature of Bayesian integration is that the relative uncertainties of an individual's prior beliefs and some external source of information should govern how the information is combined. The Bayesian approach has been successful in providing a compact description of how beliefs are updated during perceptual decision making, multisensory integration (Angelaki et al., 2009), and motor control (Ernst and Banks, 2002; Körding and Wolpert, 2004; Knill and Pouget, 2004; Summerfield and Koechlin, 2008), as well as higher-level cognitive abilities such as memory, language, and inductive reasoning (Chater et al., 2006). However, it is still unknown whether prior beliefs and social information are integrated in a Bayesian fashion that weighs the information sources by their uncertainty. How this process would be implemented in the brain is also an open question.

In this study, we tested whether people integrate social information with their prior beliefs in a Bayesian fashion and examined how the integration process is implemented in the brain. The main focus of our neural analysis is the medial prefrontal cortex; more specifically, the ventromedial (mPFC/vmPFC) and dorsomedial medial (dACC/dmPFC) subregions. The mPFC/vmPFC has a well established role in representing value estimates (Levy and Glimcher, 2012; Clithero and Rangel, 2014) and, more recently, it has been proposed that the same region tracks the reliability in these estimates (Rolls et al., 2010; De Martino et al., 2013a; Donoso et al., 2014; Barron et al., 2015; Lebreton et al., 2015). The dACC/dmPFC was chosen because of its central role in social cognition (Gallagher and Frith, 2003; Amodio and Frith, 2006; Lee, 2013; Ruff and Fehr, 2014; Wittmann et al., 2016) and, more specifically, in mediating social influence over value computation (Hampton et al., 2008; Campbell-Meiklejohn et al., 2010; Nicolle et al., 2012; De Martino et al., 2013b; Suzuki et al., 2015). However, it is unclear how social information is integrated into value computation in PFC. Does the signal in dmPFC detect a conflict between the group consensus triggering a compromise to the group evaluation? Or is it involved in a more complex Bayesian updating that takes into account variable levels of reliability in the social information as well as the level of confidence in the prior belief?

## Materials and Methods

### 

#### 

##### Participants.

Twenty-two participants 18–35 years of age [mean (SD) = 24.82 (4.10), 11 female] were recruited from the University College London (UCL) psychology subject pool. One participant was excluded because of a scanner technical problem. Another participant was excluded because of excessive head motion (>3° rotation on 4 occasions). Another 2 participants were excluded because of erratic product ratings (>3 skewness). A total of 18 participants were therefore included in the final analysis. The study was approved by the UCL Psychology Ethics Committee. Written informed consent was obtained from all participants and they were paid for participation.

##### Stimuli.

**Figure 1.**
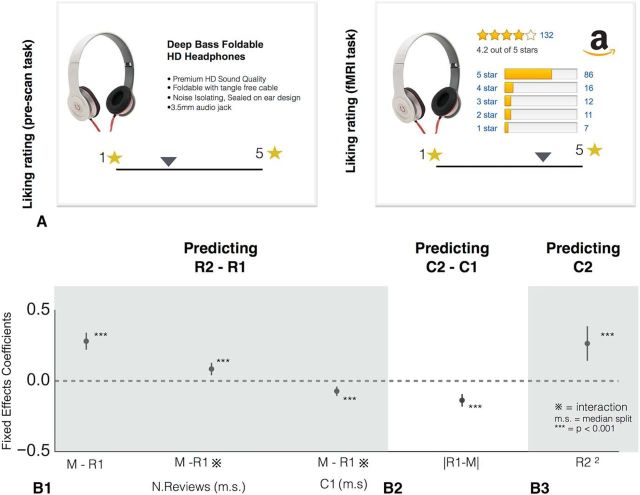
***A***, Task: In part 1 (before scanning), the participant is presented with a series of products from the retail website Amazon (e.g., headphones). The participant enters his/her liking rating *R1* followed by his/her confidence rating *C1* in the liking rating (not shown the in figure schematic above). In the part 2 (inside of the scanner), the participant sees the same item again, this time together with real reviews from the Amazon website: the mean of the reviews (1–5 stars), the number of reviewers, and a 5-bar histogram showing the distribution of ratings across reviewers. At this stage, the participant is required to enter a new liking *R2* and confidence rating *C2*. All effects predicted by the Bayesian account are significant in the appropriate direction. Shown are fixed effects coefficients from hierarchical linear regression models predicting rating update (*R2* − *R1*) (***B1***), confidence update (*C2* − *C1*) (***B2***), and second confidence rating (*C2*) (***B3***) for the following predictors: initial deviation from the group (*M* − *R1*), interaction between the initial deviation from the group and number of reviews (*M* − *R1* 
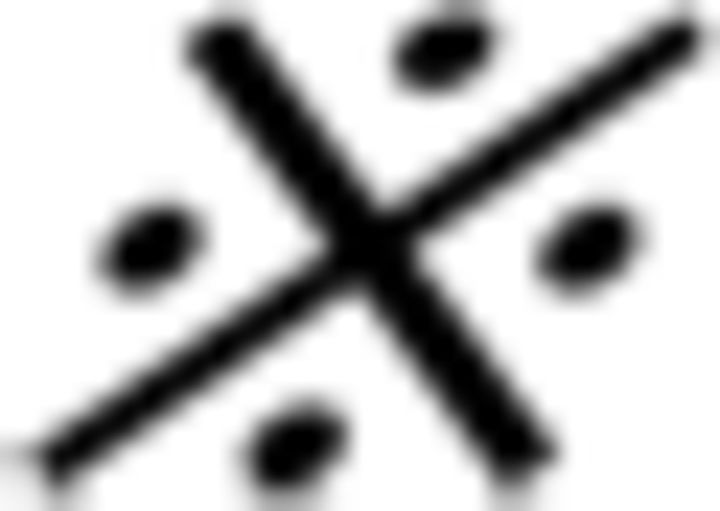
*N*), interaction between the initial deviation from the group and first confidence rating (*M* − *R1* 
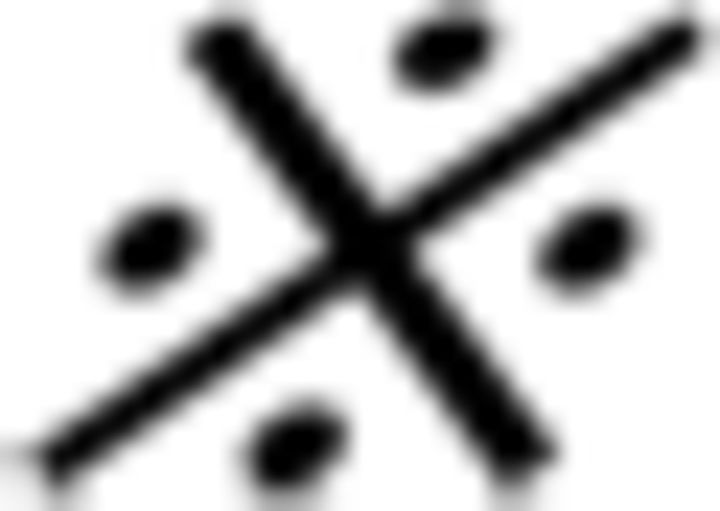
*C1*), absolute difference in a participant's initial product rating and the group consensus (|*R1* − *M*|), quadratic function of product rating (*R2*^2^). Error bars indicate 95% CI. ***p < 0.001. m.s., Median split.

Stimuli consisted of 210 pictures of products from the retail website Amazon (https://www.amazon.co.uk/) along with the product name. Each picture was presented once in each task (prescanning task and scanning task, see below) to participants in a randomized order. Four to five bullet points with descriptions of each product were provided in the prescanning task. These descriptions were based on the information available for the products on the Amazon website. During the task in the scanner, they were also presented with summary reviews of the products. This information was presented exactly as it is shown on the Amazon website: the mean of the reviews (1–5 stars), the number of reviewers, and a 5-bar histogram showing the distribution of ratings across reviewers (see Fig. 1*A*, right).

##### Prescanning task.

Participants were required to make a series of product ratings for 210 Amazon products. Participants were required to give their liking rating for each item (see Fig. 1*A*, left) and their confidence in their liking rating. A fixation cross was presented for 500 ms. Participants then moved the slider located at the bottom of the screen to indicate their rating of the product. The location of the picture of the product and the respective bullet point descriptions were left–right counterbalanced across trials. The starting position of the slider was randomized on each trial. After deciding on the product rating, the slider confirmed the selection by changing to the color red for 500 ms. Once they provided the product rating, participants were asked to indicate their confidence in their decision on a continuous sliding scale with six ticks but no numbers, with text going from “lower” to “higher” confidence. After deciding on a confidence rating, the slider confirmed the selection by changing to the color red for 1000 ms. There was no time limit for participants to rate a product or indicate their confidence rating. The 210 trials in which they did product and confidence ratings were divided into three blocks of 50 trials and one final block of 60 trials. The direction of the product rating scale and the confidence scale were reversed after two blocks of trials. If a participant started the experiment with a left to right presentation of the scales (1–5 stars and “lower” to “higher” confidence, respectively), then, after 2 blocks of trials (100 trials), participants would see the scales in right to left presentation (5–1 stars and “higher” to “lower” confidence, respectively). This was necessary to avoid visual and motor confounds during imaging in the scanning task, which is why it was preferable for participants to get accustomed to this procedure during the prescanning task. The direction of the scale for the first two blocks of trials was chosen randomly across participants. The prescanning session was conducted the same day of the scanning task.

##### Scanning task.

The scanning task presented the same 210 products that participants rated in the prescanning task. In this task, participants did not see the product descriptions. Instead, they were presented with information on other people's ratings retrieved from Amazon. In particular, the scanning task showed the number of people that rated the product, the mean rating of the product (on a scale from 1–5 stars), and the distribution of ratings. An example screen shot is provided in Figure 1*A* (right). Participants did not see their own rating from the prescanning task and were free to change their ratings in the light of other people's ratings. Participants were incentivized in this task because they were told that a product would be selected at random at the end of the experiment and would be given to them at a later date as part of their compensation. They were told that, the higher their rating for a product, the better the chances they would have in receiving that product. Products had a similar retail price range.

As in the prescanning task, a fixation cross was presented, participants decided on a product rating, and then the slider turned red for 500 ms before moving on to the confidence rating. The duration of the initial fixation cross was jittered. Unlike the prescanning task, participants were only allowed 7 s to rate a product and 4 s to report their confidence. Therefore, the timeline of the fMRI task was as follows: fixation cross (jittered between 500 and 1500 ms), item presentation + liking rating scale (7000 ms), and confidence rating (4000 ms).

##### Postscanning choice task.

At the end of the functional scans and during the structural scan, participants made 49 forced choices between a pair of products that were both rated previously during the preceding scanning task. Each pair contained one product with a low rating (randomly sampled from the bottom tercile) and one with high rating (randomly sampled from the top tercile). Participants selected the item from the top tercile on 77.29% (SD = 11.07) of the forced choices.

##### Image acquisition.

Scanning acquisition was performed using a 1.5 T Siemens TIM Avanto MRI Scanner with a 32-channel head coil used to acquire both T1-weighted structural images and T2*-weighted echoplanar images (64 × 64; 3 × 3 mm voxels; echo time, 50 ms; repetition time, 3132 ms; flip angle, 90 degrees; field of view, 192 mm) with blood oxygen level-dependent (BOLD) contrast. Each volume comprised 36 axial slices (2 mm thick). We used a specific sequence that improved the signal-to-noise ratio in the orbitofrontal cortex, a region that usually suffers from signal dropoff (Deichmann et al., 2003). To further minimize this problem, we decided to acquire the imaging data in a 1.5 T MRI scanner, which has less-pronounced dropout in this region and therefore can actually have greater BOLD sensitivity than higher-field-strength scanners (Weiskopf et al., 2006). Functional scans were acquired in four sessions, each comprising 228 volumes (∼10 min). The first five volumes in each session were discarded to allow for T1 equilibration effects. At the end of the fourth functional scan, a 5.5 min T1-weighted MPRAGE structural scan was collected, which comprised 1-mm-thick axial slices parallel to the AC–PC plane.

##### fMRI data analysis.

Image preprocessing was performed using Statistical Parametric Mapping 12 (SPM12, Wellcome Trust Centre for Neuroimaging, http://www.fil.ion.ucl.ac.uk/spm/). Image analysis was performed using SPM12. After discarding the first five dummy volumes, images were realigned to the sixth volume and unwarped using seventh-degree B-spline interpolation. Field maps were reconstructed into a single phase file and used to realign and unwarp Echo-Planar Imaging (EPI) functional images. Structural images were reregistered to mean EPI images and segmented into gray and white matter. These segmentation parameters were then used to normalize and bias correct the functional images. Normalization was to a standard EPI template based on the Montreal Neurological Institute reference brain using a nonlinear (seventh degree B-spline) interpolation. Normalized images were smoothed using a Gaussian kernel of 8 mm full-width at half-maximum.

We ran two independent general linear models (GLMs). In GLM1, onset regressors began at the presentation. Events were modeled by convolving a series of delta (stick) functions with the canonical HRF at the beginning of each item presentation. These onsets were modulated by two parametric regressors: a liking rating (*R2*) and postchoice confidence ratings (*C2*), which ranged from 0–500 on an arbitrary scale. In GLM2, onset regressors beginning at the presentation of the item were modulated by one parametric regressor: Kullback–Leibler (KL) trial-by-trial parameter estimate computed by fitting a descriptive Bayesian model to the behavioral data. Both GLMs included six movement regressors. In GLM2, two further subjects had to be excluded because the KL parameter was zero in a number of instances: this resulted in the model not being estimable in SPM. Note that the parametric regressors in both GLMs were not orthogonalized and regressors were allowed to compete to allocate the shared variance (Mumford et al., 2015). Contrast images for each regressor were tested for a significant deviation from 0 using one-sample *t* tests. Activations were reported as significant if they survived familywise error correction (FWE) for multiple comparisons across the whole brain at the cluster level. Note that the cluster-forming threshold was set as *p* < 0.001 uncorrected to ensure an a well behaved family error control (Eklund et al., 2016; Flandin and Friston, 2016). For dmPFC isolated in the GLM2, we used small-volume correction using an 8 mm sphere centered on the coordinates (−3,51,24) taken from an independent study (Hampton et al., 2008). The rfxplot toolbox (http://rfxplot.sourceforge.net/) (Gläscher, 2009) was used to extract percentage signal change at each region of interest defined by 8 mm spheres around and used for the histogram plots. Note that the signals are not statistically independent (Kriegeskorte et al., 2009) and these plots are not used for statistical inference (which was performed in the SPM framework); they are shown solely for illustrative purposes (i.e., to clarify the signal pattern in each cluster) and this has been stated explicitly in the figure legends.

##### Behavioral data analysis.

Hierarchical regression analysis were performed in R using the lme4 package (Bates et al., 2014). Participants' product (*R1* and *R2*) and confidence (*C1* and *C2*) responses were normalized (*z*-scored) separately for each participant for each of the four judgment types to correct for any potential differences in scale usage.

##### Model.

The model worked with the same *z*-scored data as used in the behavioral analyses and was fit to individual participants. First, the prior distribution (shown in blue in Fig. 4*A*) was formalized as a Gaussian distribution. For each product *j*, the mean of this distribution for participant *i* was determined by the parameter μ*_i,j_*. For the prior variance, each participant *i* had a variance parameter σ_*i*_^2^ plus a nonpositive offset parameter σ_*i*_^′^ that was included for higher confidence trials. Therefore, the prior distribution for participant *i* for product *j* is as follows:

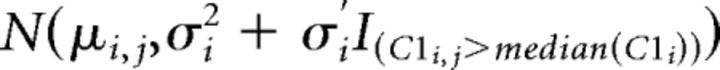
 where *I* is an indicator function returning 1 when confidence was rated above the median and otherwise 0. According to the Bayesian model, higher confidence should correspond to lower variance (i.e., greater precision). The use of the median split simplifies the model and reduces the number of assumptions needed to relate the model to the behavioral data.

The distribution of Amazon reviews for a product was also Gaussian (shown in yellow in Fig. 4*A*). The mean was fixed to *m_j_*, the observed mean of the amazon ratings for product *j*. Each participant *i* had a single parameter, τ_*i*_^2^, for the perceived variance (i.e., reliability) of the Amazon reviews in general plus two parameters related to the number of Amazon reviews. Therefore, the Amazon reviews for product *j* were parameterized to contain *v_i_* + *v_i_^′^I*_(*n_j_*>*median*(*n*))_ reviews, where *n_j_* was the number of Amazon reviews as presented to participants during the experiment and *v_i_* and *v_i_^′^* are non-negative parameters. As with confidence in the prior, this median split of the parameters by the number of reviews mirrors the behavioral analyses. A posterior distribution (shown in green in Fig. 4*A*) was then derived using Bayes theorem, so the model did not have a parameter specifically for a posterior distribution.

In summary, the model, which characterizes the degree to which participants integrate information, accounts for 420 ratings (210 initial and 210 second ratings) from each participant with 210 parameters (μ*_i,j_*) for prior means, two parameters (σ_*i*_^2^, σ_*i*_^′^) for prior variance, two parameters (*v_i_*, *v_i_^′^*) for the perceived number of Amazon reviews, and one parameter (τ_*i*_^2^) for the perceived variance in Amazon reviews. The parameter values were estimated independently for each participant to maximize likelihood of both initial and second ratings. Estimated prior mean and derived posterior mean show strong positive correlations with initial and second ratings: across 18 participants, correlation coefficients ranged from 0.75 to 0.96 [mean: 0.90, 95% confidence interval (CI): 0.88–0.93] between prior mean and an initial rating and from 0.85 to 0.96 (mean: 0.90, 95% CI: 0.89–0.92) between posterior mean and a second rating, which indicates a good fit.

Because the model was not fit to the confidence ratings, one avenue to evaluate the model is to compare the precision of its posterior to participants' second confidence ratings. Model precision should positively correlate with confidence. Correlation coefficients ranged from 0.14 to 0.40 (mean: 0.18, 95% CI: 0.12–0.24, *t*_(17)_ = 5.95, *p* < 0.001). The main justification for the basic approach (i.e., integrating prior and likelihood information according to their uncertainties) comes from the behavioral results reported below.

Using the estimates from the model, the degree of resistance to Amazon reviews is computed for each participant for each product as follows:

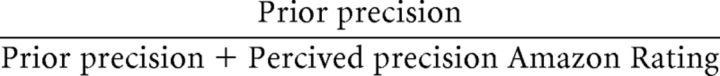
 where prior precision is the inverse of prior variance and perceived precision of Amazon rating is estimated Amazon N divided by estimated Amazon variance. Given Bayes theorem, the above specification captures how heavily prior mean is weighted toward posterior mean.

Specifically, the degree of resistance to Amazon reviews is 1 when Amazon rating is completely ignored and prior mean is the same as posterior mean. In addition, the degree of resistance to Amazon reviews to is 0 when prior is completely discarded and Amazon mean is the same as posterior mean. A larger value indicates that Amazon mean is more heavily weighted toward posterior mean than prior mean is.

This degree of resistance to Amazon reviews was mean averaged for each participant across 210 product ratings.

## Results

We developed a task in which participants were presented with a series of products from the retail website Amazon (e.g., headphones, USB pens, mugs). Participants were required to give their initial liking rating (*R1*) for each item and their confidence (*C1*) in their liking rating (Fig. 1*A*). Both measures were collected before scanning. In the second part of the experiment, we recorded the participants' neural activity (using fMRI) while they were shown each item again, this time together with reviews from other customers who had bought those products (these were the real reviews from the Amazon website). This information was presented as it is shown on the Amazon website: the mean of the reviews (1–5 stars), the number of reviewers, and a 5-bar histogram showing the distribution of ratings across reviewers (Fig. 1*A*). At this second stage, we elicited another liking rating (*R2*), again followed by a new confidence rating (*C2*).

To foreshadow the results, people followed the basic tenets of Bayesian integration. A descriptive Bayesian model consistent with these behavioral results made it possible to conduct a trial-by-trial fMRI analysis to isolate brain regions that tracked the degree to which social information and its reliability affected participants' beliefs.

### Behavioral results

The central behavioral question was whether participants' initial product rating (*R1*) was combined with the Amazon group mean (*M*) in a Bayesian fashion to yield an updated product rating (*R2*). The key property of Bayesian integration is weighting information by its reliability, which here corresponds to updating more toward the group consensus when initial confidence is low and the group is large. To evaluate whether people's judgments were consistent with Bayesian integration, we conducted a series of hierarchal regression analyses to assess which sources of information people considered when rating the products.

In particular, we performed a hierarchical regression analysis to isolate the factors that contributed to the update from the first to second product rating (i.e., *R2* − *R1*). The first analysis considers whether people conform to the group mean, which in itself does not indicate Bayesian integration. We found that participants' initial deviation from the group (i.e., *M* − *R1*) was a reliable positive predictor of participants' update χ_(2)_^2^ = 1000.79, *p* < 0.001), meaning that participants systematically updated their initial liking ratings in the direction of the group consensus (expressed here by the mean reviews). More complex regression models included additional terms that evaluated whether participants' judgments were consistent with aspects of Bayesian integration. In particular, interactions terms including confidence and the number of reviews were also assessed using median splits. Median splits were used because the psychological scaling of these quantities is unlikely to be linear. These scaling issues, which are topics of investigation in their own right (Siegler and Opfer, 2003; Kvam and Pleskac, 2016), are beyond the scope of this contribution.

Consistent with Bayesian updating, the magnitude of movement toward the group ratings was modulated by the level of confidence in their first rating such that, when the initial confidence was low, participants were more strongly influenced by the group consensus (negative interaction between *M* − *R1* and median split on initial confidence *C1* χ_(2)_^2^ = 15.62, *p* < 0.001). This result is consistent with half of the Bayesian integration account, namely that participants' uncertainty in their own beliefs guides their judgments. Evaluating the other half of the Bayesian account, the update toward the group consensus (mean of the Amazon's reviews) was largest when that information was more reliable because the number of reviews was higher (positive interaction between *M* − *R1* and median split of number of reviews; χ_(2)_^2^ = 24.33, *p* < 0.001; Fig. 1*B*). Finally, we found that the full regression model, which simultaneously takes into account both sources of uncertainty, was superior to regressions that were only sensitive to either confidence or number of reviews (χ_(2)_^2^ = 17.55, *p* < 0.001, and χ_(2)_^2^ = 26.25, *p* < 0.001), respectively. In summary, the change in rating from *R1* to *R2* was consistent with Bayesian integration.

According to a Bayesian account of integration, confidence should be highest in the second rating when the initial rating and the group mean align. Indeed, the overall confidence decreased (i.e., *C2* − *C1*) when the absolute difference in a participant's initial product rating and the group consensus (i.e., |*R1* − *M*|) was high (χ_(2)_^2^ = 36.79, *p* < 0.001) and confidence elicited after the second rating (*C2*) was a quadratic function of product rating (*R2^2^*); that is, that confidence was highest for products at the ends of the rating scale (χ_(2)_^2^ = 547.92, *p* < 0.001).

Together, these analyses established that participants integrated their initial impression of a product and the group consensus by taking into account the uncertainty associated with each source of information (Fig. 1*B*).

### fMRI results

**Figure 2.**
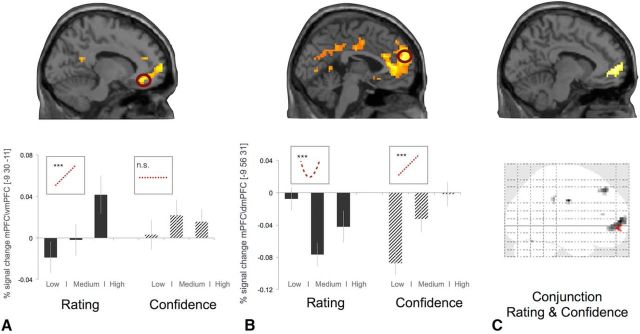
***A***, BOLD signal in mPFC/vmPFC correlates with monotonic increase in liking ratings (peak= −9,38,−11 mm, *z* = 4.21, *p* < 0.05, FWE corrected at cluster level). For illustration purposes only, percentage signal change in vmPFC (8 mm sphere centered at the peak of the main effect −9,38,−11) for 3 levels or rating level and confidence (low, medium, and high) are shown; a linear relation between percentage signal changes and rating level and a nonsignificant (linear or quadratic) relation between percentage signal changes and confidence level. ***B***, Activity in mPFC (extending in vmPFC and dmPFC) tracked monotonically the increases in confidence ratings (peak = −9,56,31, *z* = 4.55, p < 0.05, FWE corrected at cluster level). For illustration purposes only, percentage signal change in mPFC/dmPFC (8 mm sphere centered at the peak of the main effect −9, 56, 31) for 3 levels or rating and confidence (low, medium, and high) are shown; a linear relation between percentage signal change and confidence levels and a significant quadratic relation between percentage signal change and rating levels. The histogram plots are not used for statistical inference (which was performed in the SPM framework); they are shown solely to illustrate the dynamic of the BOLD signal. Error bars indicate SEM. SPM maps are thresholded at *p* < 0.005 uncorrected for display purposes. ***C***, Conjunction analysis for rating and confidence: activity in mPFC/vmPFC (peak activation at −12,59,4, *z* = 3.61, *p* < 0.05, small volume corrected at peak level using at 8 mm centered at −2,52,−2 from Lebreton et al., 2015).

We tested how the brain represents the value assigned to each item and the confidence in that value. We constructed a GLM (GLM1), in which each trial was modulated by two parametric regressors: liking rating *R2* and confidence *C2* (in the liking rating) both collected during the scanning (see Materials and Methods for more details). Consistent with previous work (for meta-analyses, see Clithero and Rangel, 2014), we show that activity in mPFC/vmPFC responded linearly to increasing levels of subjective liking rate (*p* < 0.05, FWE corrected at cluster level; cluster-forming threshold *p* < 0.001; see Materials and Methods for more details; Fig. 2*A*). In the same analysis, we show that mPFC also tracked subjective levels of confidence (*p* < 0.05, FWE corrected at cluster level; cluster-forming threshold *p* < 0.001; Fig. 2*B*). To test whether liking rating and confidence in the liking rating were encoded in the same brain region, we performed a conjunction analysis between liking rating and confidence. This analysis isolated a functional cluster in mPFC/vmPFC (peak activation at −12, 59, 4, *z* = 3.61, *p* < 0.05, small volume corrected at peak level using at 8 mm centered at −2,52,−2 from Lebreton et al., 2015; Fig. 2*C*). This result is consistent with the recent finding that response in the same cluster in mPFC/vmPFC represents both a linear response to pleasantness rating and a quadratic explanation of pleasantness rating that, in that study, was used as a proxy for confidence. (Lebreton et al., 2015).

**Figure 3.**
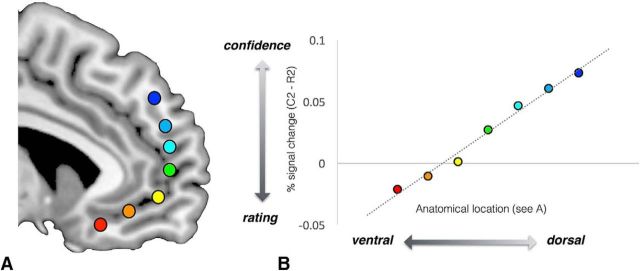
Spatial gradient analysis along the ventral–dorsal axis of mPFC (see colored dots) for a contrast between the parametric response to rating and the parametric response to confidence (*R2* − *C2*). Data from seven anatomical locations (***A***) are mapped onto a line and the spatial regression slope is computed (***B***). Across participants, there is a robust gradient along the medial lane of PFC with response to rating expressed in the more ventral part and response to confidence represented in in the more dorsal part.

We then tested whether there existed a mPFC gradient coding for confidence and value along the ventral–dorsal axis. We fitted a hierarchical linear regression model to contrast confidence versus rating (*C2* − *R2*) extracted from 7 different locations (signal extracted by 8 mm sphere for each location) along the mPFC (Fig. 3*A*). These locations were selected solely on an anatomical basis as opposed to by peak activity from any preceding analysis. Across the group, we found a significant gradient along the rating/confidence axis (slope = 0.02, *t*_(20.95)_ = 9.17, *p* < 0.0001). To confirm that the gradient was driven by both rating and confidence, we performed two more regression analyses, which revealed a negative ventromedial gradient in BOLD activity in response to rating (slope = −0.01, *t*_(27.06)_ = 4.74, *p* < 0.0001) and a positive ventromedial gradient in BOLD activity in response to confidence (slope = 0.01, *t*_(17.80)_ = 7.05, *p* < 0.0001).

**Figure 4.**
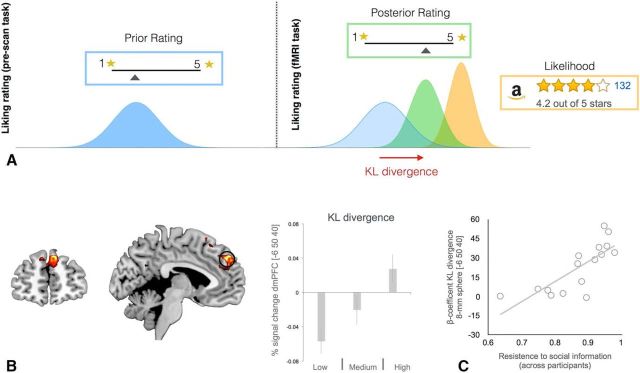
***A***, Schematic representation of the Bayesian update of liking ratings in response to social information communicated through reviews. The KL divergence parameter indexes the impact of the reviews in shifting the liking rate from the first rating (made in the absence of review information) and the second rating (performed by the participants after seeing the Amazon reviews). ***B***, BOLD signal in dmPFC (peak = −6,50,40) correlates with increase in KL divergence (*z* = 3.66, *p* < 0.05, FWE small volume corrected). Percentage signal change for three levels (low, medium, and high) of KL divergence is shown. The histogram plot is not used for statistical inference (which was performed in the SPM framework); it is shown solely to illustrate the dynamic of the BOLD signal. Error bars indicate SEM. ***C***, Between-subject correlation between activity in dmPFC (8 mm ROI centered at −6,50,40) and the degree of resistance to social information (*r* = 0.77, *p* < 0.0005). This analysis shows that people who are less influenced by the opinion expressed by others in the reviews have overall more activity in this area.

To quantify how social information shapes the value representation in PFC, we developed a Bayesian model (a model schematic overview of the Bayesian model is shown in Fig. 4*A*; see Materials and Methods for full detail). The Bayesian model aimed to explain the value update with three steps: (1) an initial rating is drawn from a prior distribution, (2) this prior distribution is updated in the light of Amazon reviews to form a posterior distribution, and (3) a second rating is drawn from the posterior distribution.

The Bayesian model allowed us to calculate how social information influenced participants' initial impressions of value. In the Bayesian framework, the KL divergence can quantify the extent to which a prior distribution is updated to form a posterior distribution (Fig. 4*A*). Therefore, a larger KL divergence indicates a greater preference update. KL divergence is critical in the fMRI analyses because it provides a combined measure of trial-by-trial update that takes into account both the uncertainty reflected by the participant's confidence rating and the number of reviews (i.e., group size). Letting *p* and *q* denote prior and posterior density function, respectively, KL divergence is computed as follows:

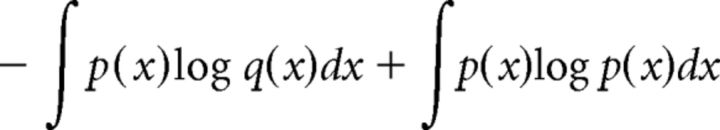
 In our Bayesian model, both prior and posterior distributions were Gaussian distributions. Therefore, the above equation reduces to the following:

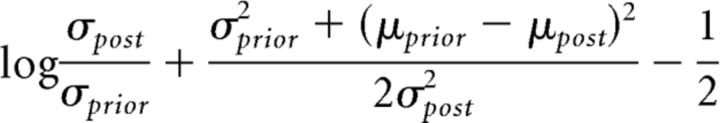
 where μ_prior_ and μ_post_ are the prior and posterior means and σ_*prior*_^2^ and σ_*post*_^2^ are the prior and posterior variances.

The Bayesian model enables a key analysis, namely the identification of brain areas that track the magnitude of Bayesian value update in the presence of social information. A new GLM (GLM2) was constructed using a parametric regressor that tracked the trial-by-trial KL divergence estimates using the aforementioned model fits. KL divergence takes into account all aspects of belief change such as the initial rating and confidence and the mean and number of Amazon reviews. This analysis found a trial-by-trial response in dmPFC (Fig. 4*B*) to parametric increases in KL divergence (*p* < 0.05 small volume corrected centered on a priori hypothesized coordinates −3,51,24 from Hampton et al., 2008). In other words, activity in this cluster indexes the size of update of a value judgment after the social information provided by the Amazon review has been presented.

We then tested whether this same region indexed how likely participants were to conform to the social consensus in general. We constructed a between-subjects measure of how resistant subjects were to the social information carried by the reviews. Specifically, the degree of resistance to Amazon reviews is 1 when Amazon rating is completely ignored and prior mean is the same as posterior mean. In addition, the degree of resistance to Amazon reviews is 0 when prior is completely discarded and Amazon mean is the same as posterior mean. A larger value indicates that Amazon mean is more heavily weighted toward posterior mean than prior mean (see Materials and Methods for more details). We then extracted the BOLD signal in this region of interest (8 mm sphere centered at the peak of the effect isolated from the independent within-subject analysis GLM2) and tested whether the activity in this region showed a positive modulation by the individual ability to resist to the social information (carried by the reviews showed by Amazon website) while constructing their value judgments. This analysis showed that activity in this cluster of dmPFC (Fig. 4*B*) was higher for those individuals who were less influenced by the information carried by the reviews of other people (*r* = 0.77, *p* < 0.0005). This between-subject analysis and the preceding trial-by-trial within-subject analysis provide complementary viewpoints on dmPFC's role in belief updating.

## Discussion

In this study, we show that the degree to which value and confidence judgments are influenced by the opinions of others (expressed through online reviews) is modulated by both the reliability of the group's opinions and the individual's confidence in their own prior belief. We found that people's updated judgments were consistent with a Bayesian integration account that updated more toward the group consensus when initial confidence was low and the group was large. The model was verified by eliciting liking and confidence judgments twice, the first time when each item was presented in isolation and a second time when it was presented together with the reviews collected from the Amazon website. At the behavioral level, we found that the number of reviews significantly modulated the shift toward the group consensus (i.e., toward the mean of the Amazon's reviews). This shift was more substantial when the participants were less sure in their initial ratings (low level of confidence) and a large shift toward the group consensus was characterized by a drop in the overall level of confidence. These results showed that uncertainty in both the social information and participants' initial estimates (gauged through confidence reports) modulated the participants' behavioral responses.

To help quantify the impact of the social information on the computation of value and confidence, we constructed a simple Bayesian model that captured the main aspects of the behavioral results. Although not fitted to the confidence data, the model correctly predicted confidence as evidenced by a positive correlation between the precision of its posterior distributions with confidence collected during the scanning phase. This finding is consistent with the idea that verbal reports of confidence closely match the formal concept of precision as defined in Bayesian probability (Meyniel et al., 2015a, 2015b; but see Pouget et al., 2016).

Analysis of the fMRI data showed that mPFC/vmPFC tracked both the subjective rating and the confidence level in that estimate. Our work adds to recent studies that have considered the role that these areas play in representing confidence during value-based choice. For example, De Martino et al. (2013a) have shown that activity in vmPFC correlates with both difference in value and confidence in a binary choice task. Our study provides a strong test of this characterization of the vmPFC because participants judged objects in isolation rather than in a binary choice task, which resulted in rating and confidence sharing a quadratic as opposed to linear relationship (i.e., confidence is highest for extreme ratings). Nevertheless, we found that vmPFC tracked both the participants' confidence and liking ratings. These findings are consistent with a recent study by Lebreton et al. (2015) finding that activity in mPFC/vmPFC correlates with both the linear and quadratic expansion of the pleasantness ratings that might reflect an automatic assessment of confidence.

We helped to resolve the relationship between confidence and value representations in the PFC by finding a smooth gradient along the medial ventral–dorsal axis of PFC with liking ratings manifested more ventrally and confidence ratings more dorsally. A possible interpretation of this result is that there are two populations of neurons distributed along the ventral–dorsal axis of mPFC, with the more ventral region coding for the mean value estimate and the more dorsal region coding for the reliability of these estimates (either measured directly by confidence ratings or indirectly through the quadratic expansion of liking rating). A similar gradient has been found for values that are executed (represented more ventrally) and values that are modeled but not executed (represented more dorsally; Nicolle et al., 2012). An intriguing possibility is the more dorsal part of the PFC is implicated in a high-order belief inference (Yoshida and Ishii, 2006) for monitoring the reliability of the behavioral strategy in which the agent is currently engaged (Donoso et al., 2014), as in value estimation in our study. Such inferences may tap similar processes with those used to reason about other people's states, which is also hypothesized to involve the more dorsal regions of PFC (Denny et al., 2012).

Our modeling approach quantified the degree of value update resulting from exposure to the social information carried by the reviews on a trial-by-trial basis. In our model, the KL divergence indexes the shift from the before posterior belief when new evidence (i.e., likelihood) is available. Our model-based fMRI analysis showed that activity in dmPFC positively correlated with KL divergence. We found that the dmPFC responded at the trial-by-trial level to the size of update in value judgment from the prior judgments (made in absence of social information) to posterior judgments after the participants were exposed to other people's opinions (expressed at the aggregate level by the reviews). Recent work using a perceptual decision-making task also found that activity in dmPFC (though slightly more posterior to the peak of our main activation) covaried with belief updating in response to new information (O'Reilly et al., 2013).

Earlier work implicates the dmPFC in theory of mind and in social cognition more generally (Amodio and Frith, 2006; Behrens et al., 2009) through enabling agents to take into account the judgments of others during value-based choice (Behrens et al., 2008; Hampton et al., 2008; Behrens et al., 2009; Coricelli and Nagel, 2009; De Martino et al., 2013b; Suzuki et al., 2015). Although these studies focused on the dmPFC, related studies have found a role for other brain regions in the social modulation of learning and hedonic experience. For example, the rostral cingulate cortex and striatum have been found to track the mismatch between the opinions of an individual and a group (Klucharev et al., 2009). This basic mismatch is analogous to deviating from the group in our study absent weighting by the reliability of the individual and group information sources. A second fMRI study investigated how teenagers were influenced by popularity ratings in judging song tracks (Berns et al., 2010). Their analyses (using a masking procedure) focused on a network of regions (including insula) that were activated during hedonic experience (i.e., listening to the song track), which can be contrasted with the more abstract evaluation processes invoked by our task.

From a computational perspective, internal models should be updated when new information (or a change in the task) makes the current model inadequate (Durstewitz et al., 2010; Domenech and Koechlin, 2015). This shift usually pushes the agent toward more explorative behaviors (Daw et al., 2006; Hayden et al., 2011; Karlsson et al., 2012; O'Reilly et al., 2013). Other studies have shown that activity in dmPFC tends to increase in those situations in which an agent has to abandon the current model (because it has become unreliable) and initiate exploration (Karlsson et al., 2012; O'Reilly et al., 2013; Tervo et al., 2014). One possibility is that the update is triggered by noradrenaline (Yu and Dayan, 2005) that signals a mismatch between the predictions of the current internal model and external feedback (Yu and Dayan, 2005; Payzan-LeNestour et al., 2013; McGuire et al., 2014). A recent study has provided experimental support for this idea by showing that noradrenaline mediates this switch by changing the noradrenergic inputs to the ACC (Tervo et al., 2014).

Our results suggest that dmPFC involves a higher-order inference similar to that required when estimating the reliability in one's own appraisals of value (see also Nicolle et al., 2012). It is possible that, in most social interactions, humans are required to represent others' preferences (an ability linked to theory of mind) and that this information is used to update their own preferences. An intriguing possibility is that the basic computation of dmPFC is to represent and manipulate multiple beliefs, explaining its prominent role in theory of mind.

Finally, although, at the within-participants level, dmPFC activity and KL divergence positively correlated, at the between-participants level, we found that activity in dmPFC in response to KL divergence was more pronounced for people less amendable to conforming to the group consensus (i.e., adjusting their ratings toward the group's ratings). This result is consistent with dmPFC playing a role in monitoring differences between an individual's opinion and that of the group. Greater dmPFC involvement overall appears to indicate heightened sensitivity to divergence with the group, which may facilitate an individual maintaining their original opinion to a greater extent. In contrast, a person who readily conforms to the group consensus would not integrate personal beliefs with the group's as much as wholesale accept the group's opinion. In such a case, the dmPFC should not be very active overall, assuming its role is to monitor differences between belief representations. In reality, people should fall along a continuum of conformity such that dmPFC activity tracks both trial-by-trial updates and the overall propensity to conform. These findings are also consistent with two recent TMS studies finding that that stimulating posterior medial frontal cortex modulates social conformity (Klucharev et al., 2011) and choice-induced preference changes (Izuma et al., 2015).

In conclusion, our work suggests that the update of value and confidence in response to social information involves an integration mechanism analogous to that used in perceptual decision making. Belief update follows Bayesian principles in which clear signatures of value, confidence, and belief update are reflected in PFC activity.
